# Layer 5 Callosal Parvalbumin-Expressing Neurons: A Distinct Functional Group of GABAergic Neurons

**DOI:** 10.3389/fncel.2018.00053

**Published:** 2018-03-06

**Authors:** Hector Zurita, Paul L. C. Feyen, Alfonso Junior Apicella

**Affiliations:** Department of Biology, Neurosciences Institute, University of Texas, San Antonio, San Antonio, TX, United States

**Keywords:** long-range, GABAergic neurons, interneurons, callosum, interhemispheric connectivity, parvalbumin, layer 5, Kv1.1 potassium channel

## Abstract

Previous studies have shown that parvalbumin-expressing neurons (CC-Parv neurons) connect the two hemispheres of motor and sensory areas via the corpus callosum, and are a functional part of the cortical circuit. Here we test the hypothesis that layer 5 CC-Parv neurons possess anatomical and molecular mechanisms which dampen excitability and modulate the gating of interhemispheric inhibition. In order to investigate this hypothesis we use viral tracing to determine the anatomical and electrophysiological properties of layer 5 CC-Parv and parvalbumin-expressing (Parv) neurons of the mouse auditory cortex (AC). Here we show that layer 5 CC-Parv neurons had larger dendritic fields characterized by longer dendrites that branched farther from the soma, whereas layer 5 Parv neurons had smaller dendritic fields characterized by shorter dendrites that branched nearer to the soma. The layer 5 CC-Parv neurons are characterized by delayed action potential (AP) responses to threshold currents, lower firing rates, and lower instantaneous frequencies compared to the layer 5 Parv neurons. Kv1.1 containing K^+^ channels are the main source of the AP repolarization of the layer 5 CC-Parv and have a major role in determining both the spike delayed response, firing rate and instantaneous frequency of these neurons.

## Introduction

The local connectivity of the cerebral cortex comprises a diverse set of GABAergic neurons distinguishable by intrinsic physiological properties, dendritic fields, and axonal connections. These properties define the functional task of each respective sub-type in the balance of excitation and inhibition within cortical circuits (for review see, Isaacson and Scanziani, 2011). Although the existence of long-range GABAergic neurons has been proven anatomically (Seress and Ribak, 1983; Ribak et al., 1986; Toth and Freund, 1992; Toth et al., 1993; Freund and Buzsáki, 1996; for review see Caputi et al., 2013; Tremblay et al., 2016), previous studies have primarily focused on the local cortical circuit organization of GABAergic interneurons (Buzsáki, 1984; Ali et al., 1999; Holmgren et al., 2003; Maccaferri and Lacaille, 2003; Pouille and Scanziani, 2004; Silberberg and Markram, 2007; Pouille et al., 2009, 2013; Stokes and Isaacson, 2010; Hayut et al., 2011; Pfeffer et al., 2013; Crandall and Connors, 2016), and inhibition is frequently described as being exclusively local. While much progress has been made in classifying local and long-range excitatory projections that compose the canonical cortical circuit organization, few studies have contributed to our understanding of the cortical organization of long-range GABAergic neurons. The functional relevance of this class of GABAergic neurons and how different subtypes play distinct roles in cortical processing by virtue of differences in their morphology, electrophysiology, molecular content, and synaptic connectivity has not been determined.

Anatomical studies have suggested that about 1%–10% of the cortical GABAergic neurons in rodents, cats and monkeys are characterized by long-range cortical projections (McDonald and Burkhalter, 1993; Tomioka et al., 2005; Higo et al., 2007, 2009; Tomioka and Rockland, 2007). In addition, these studies have demonstrated the presence of callosal projections emanating from non-pyramidal neurons (Code and Winer, 1985; Hughes and Peters, 1990, 1992; Peters et al., 1990; Gonchar et al., 1995). Interestingly, a fraction of the axons of the corpus callosum, the major pathway for the transfer of sensory and motor information between the two hemispheres, are immunoreactive for the enzyme involved in the synthesis of the GABA neurotransmitter (Fabri and Manzoni, 2004), suggesting long-range interhemispheric inhibition occurs in adult brains.

Indeed, results from our laboratory and others (Jinno and Kosaka, 2004; Lee et al., 2014; Rock et al., 2017) demonstrated that parvalbumin-expressing neurons contribute to long-range GABAergic projections. A large proportion of parvalbumin-expressing neurons spanning from layers 2/3 to layer 6 project through the corpus callosum and connect the two hemispheres of the auditory, visual and motor cortex (Rock et al., 2017). This suggests that callosal long-range parvalbumin-expressing neurons are a general feature of the neocortical circuit’s organization. Like other parvalbumin-expressing cortical neurons, CC-Parv neurons also provide local inhibition onto nearby pyramidal neurons and receive thalamocortical input (Rock et al., 2017). These results provided further evidence of the heterogeneity of long-range neocortical GABAergic projections (Tomioka et al., 2005; Higo et al., 2007, 2009; Tomioka and Rockland, 2007; McDonald et al., 2012; Melzer et al., 2012; Lee et al., 2014; Rock et al., 2016), suggesting that different group of GABAergic neurons can have distinct functional role in the cortical network with respect to their molecular, morphological, electrophysiological and synaptic properties. However, to date, no in-depth anatomical and electrophysiological characterization of layer 5 long-range projecting GABAergic neuron in the mouse auditory cortex (AC) or any other cortical area exists.

The present study focused on three main goals: (1) determine if layer 5 parvalbumin-expressing neurons that send long-range GABAergic projections to the contralateral cortex via the corpus callosum have different dendritic fields compared to the rest of layer 5 parvalbumin-expressing neurons; (2) describe the excitability properties of these layer 5 corticocortical parvalbumin-expressing neurons (CC-Parv); and (3) determine the molecular mechanisms responsible for the excitability properties of these layer 5 CC-Parv neurons. Our approach consisted of viral tracing, anatomical and electrophysiological methods to investigate these questions.

We found that layer 5 parvalbumin-expressing neurons have distinct morphologies, with the layer 5 CC-Parv neurons having an extensive dendritic arborization compared to the layer 5 Parv neurons. In addition, layer 5 CC-Parv neurons are characterized by a delayed action potential (AP) response near-threshold and a lower instantaneous frequency. The presence of Kv1 channels are the main sources of the repolarization of the CC-Parv AP and have a major role in determining both the spike delayed response, firing rate and instantaneous frequency observed in these neurons. In sum, we describe two layer 5 sub-classes of parvalbumin-expressing neurons, with findings suggesting separate functions in cortical circuits and differential engagement during interhemispheric communication such as cortical oscillations.

## Materials and Methods

All animal procedures were approved by the Institutional Animal Care and Use Committee at the University of Texas at San Antonio. Procedures followed animal welfare guidelines set by the National Institutes of Health. Mice used in this experiment were housed in a vivarium maintaining a 12 h light/dark schedule and given *ad libitum* access to mouse chow and water.

### Transgenic Mouse Lines

The following mouse lines were used in this study:

Parv-Cre: B6;129P2-Pvalb*^tm1(cre)Arbr^*/J, The Jackson Laboratory stock number 008069; ROSA-tdTomato reporter: B6.CG.Gt(ROSA)26Sor*tm14(CAG-tdTomato)Hze*/J, The Jackson Laboratory, stock number 007914. Parv-Cre female mice were crossed with a ROSA-tdTomato reporter male mouse to generate a Parv-Cre-tdTomato line (parvalbumin-containing neurons expressed both Cre and tdTomato).

### Stereotaxic Injections

#### Basic Surgical Procedures

Mice were initially anesthetized with isoflurane (3%–5%; 1 L/min O_2_ flow) in preparation for the stereotaxic injections detailed in the sections below. The mice were head-fixed on a stereotaxic frame (Model 1900; Kopf Instruments) using non-rupture ear bars. Anesthesia was maintained at 1%–1.5% isoflurane for the duration of the surgery. A warming pad was used to maintain body temperature during the procedure. Standard aseptic technique was followed for all surgical procedures. Injections were performed using a pressure injector (Nanoject II; Drummond Scientific) mounted on the stereotaxic frame. Injections were delivered through a borosilicate glass injection pipette (Wiretrol II; Drummond Scientific) with a taper length of ~30 mm and a tip diameter of ~30 μm. After lowering it to the target injection depth, the glass pipette remained in place 5–10 min both before and after the injection was made. Both male and female Parv-Cre or Parv-Cre-tdTomato mice, P35–42 at the time of the injection, were utilized in these experiments.

#### Retrograde Labeling of CC-Parv Neurons

CC-Parv neurons in the AC were retrogradely labeled with GFP using AAV1.CAG.Flex.eGFP.WPRE.bGH (AAV1.GFP.Flex; University of Pennsylvania Vector Core) stereotaxically injected into the right AC of Parv-Cre-tdTomato mice (20 mice from 12 litters). Injections were performed as above, with the following parameters: Stereotaxic coordinates for the AC injection site were 2.50 mm posterior and 4.35 mm lateral to Bregma. Approximately 40 nanoliter of AAV1.GFP.Flex was delivered between two depths in the AC, 1.0 mm and 0.75 mm below the surface of the brain, over the course of 5 min.

#### *In Vitro* Slice Preparation and Recordings

Slice preparation and electrophysiological recordings were performed as previously described (Rock and Apicella, 2015; Rock et al., 2016, 2017). We allowed 12–24 days for expression of GFP. The brains of the animals were dissected and sectioned in a chilled cutting solution (in mM: 110 Choline Chloride, 25 NaHCO_3_, 2.5 KCl, 1.25 NaH_2_PO_4_, 0.5 CaCl_2_, 7 MgSO_4_, 25 D-glucose, 11.6 Sodium Ascorbate, 3.1 Sodium Pyruvate). Coronal slices containing the primary AC (300 μm, Bregma −2.2 to −3.1) were made using a vibratome (Leica VT1200S, Leica Biosystems). Slices were incubated in oxygenated artificial cerebral spinal fluid (ACSF solution in mM: 126 NaCl, 2.5 KCl, 26 NaHCO_3_, 2 CaCl_2_, 1 MgCl_2_, 1.25 NaH_2_PO_4_, 10 D-glucose in a submerged chamber at 35–37°C for 30 min and then at room temperature (21–25°C) until used for recordings.

Viral-retrograde-labeled CC-Parv neurons were located in the AC contralateral to the injection site in the AC. The AC was identified as previously described. We also used two landmarks similar to the ones used in a previous study (Oviedo et al., 2010; Rock and Apicella, 2015; Rock et al., 2016, 2017). Briefly, we centered the *x*-axis on the boundary between the dorsal and ventral division of the lateral geniculate body, then a perpendicular line, *y*-axis, was drawn using custom software to align the AC from mouse to mouse.

Whole cell recordings were performed at 31–33°C in ASCF solution using pipettes with 3–4 MΩ resistance. Intrinsic properties were recorded using a K-based intracellular solution (in mM: 20 KCl, 120 potassium gluconate, 10 HEPES, 0.2 EGTA, 4 ATP, 0.3 GTP, 10 mM phosphocreatine, and either 0 or 0.3%–0.5% biocytin). The pharmacological blockers used were CPP (5 μM, Tocris), NBQX (10 μM, Abcam), gabazine (10 μM, Abcam), DTX-I and DTX-K (50 and 100 nM, Alomone labs). The software program Ephus (Suter et al., 2010,^1^) was used for hardware control and data acquisition. Signals were filtered at 4 kHz and sampled at 20 kHz. Pipette capacitance was compensated for and the series resistance during recordings was lower than 20 MΩ. The resting membrane potential (*V*_m_) was calculated in current-clamp mode (*I* = 0) immediately after breaking in. Series (*R*_s_) and input resistance (*R*_in_) were calculated in voltage-clamp mode (*V*_hold_ = −70 mV) by giving a −5 mV step, which resulted in transient current responses. *R*_s_ was determined by dividing the voltage step amplitude by the peak of the capacitive current generated by the voltage step. The difference between baseline and steady-state hyperpolarized current (Δ*I*) was used to calculate *R*_in_ using the following formula: *R*_in_ = −5 mV/Δ*I* − *R*_s_. Subthreshold and suprathreshold membrane responses in current-clamp were elicited by injecting −100 to +500 pA in 50 pA increments while holding the baseline membrane potential at −70 mV with an injection of the appropriate amount of current. The first resulting AP at rheobase (the minimal current of infinite duration (experimentally limited to 1 s required to generate an AP) was analyzed for AP width. The adaptation ratio was measured at the current step that gave the closest APs firing rate to 20 Hz. Adaptation ratio was calculated dividing the first instantaneous frequency by the last (f_2_/f_last_). Afterhyperpolarization (AHP) was characterized by the AHP trough, duration, and area. AHP trough is the difference between threshold and minimum AHP. AHP duration is the time defined by the AP threshold membrane potential. AHP area is the area under a line defined by the AP threshold membrane potential.

#### Histology

During whole-cell recordings, neurons were filled with an internal solution containing 0.3%–0.5% biocytin. Filled neurons were held for at least 30–50 min, and then the slices were fixed in a formalin solution at 4°C until ready for processing. The slices were washed six times in PBS and placed in a 4% streptavidin (Alexa Fluor 680; Life Technologies) solution with 0.3% Triton X-100 in PBS. Slices were allowed to incubate in this solution at 4°C overnight, then washed six times in PBS and mounted with Fluoromount-G on a glass microscope slide. Confocal images were taken with a Zeiss LSM-710 microscope at varying magnifications (3–63× objective). Individual high magnification images were stitched together, when necessary. Image adjustment was performed in ImageJ for brightness/contrast corrections and pseudocoloring. Neurons were morphologically reconstructed in three dimensions using the Simple Neurite Tracer plugin for ImageJ (Longair et al., 2011).

#### Morphological Quantification

Individual high magnification images of layer 5 Parv and CC-Parv neurons were stitched together, when necessary, using XuvStitch software (Emmenlauer et al., 2009). Images were rotated, cropped and the brightness/contrast was adjusted in ImageJ. The dendrites of these neurons were morphologically reconstructed in three dimensions using the Simple Neurite Tracer plugin for ImageJ. Cell morphological measurements, including calculation of soma area, dendritic length, number of branches and Sholl analysis (Sholl, 1953), were performed using the Simple Neurite Tracer plugin (Longair et al., 2011; Ferreira et al., 2014) and region of interest measurement tool in ImageJ.

### Data Analysis

Error bars in all figures represent S.E.M. Data and statistical analysis was performed offline using MATLAB routines (MathWorks). Group data represent the mean ± S.E.M. Group comparisons were made using a Student’s *t*-test if the data were normally distributed (assessed with Lilliefors’ test) or the rank-sum for non-normally distributed data, with significance defined as *p* < 0.05.

## Results

### Morphological Properties of Layer 5 Long-Range Callosal Parvalbumin-Expressing Neurons in the Mouse AC

**Figure 1:**
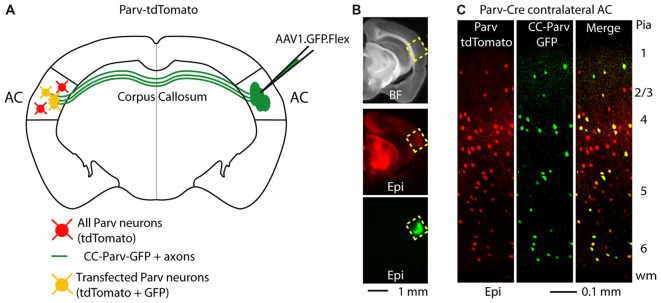
Callosal parvalbumin-expressing neurons in the mouse auditory cortex (AC). **(A)** Schematic depicting injection site using the Parv-Cre-tdTomato mouse line to retrogradely transfect CC-Parv neurons in the contralateral AC with GFP. **(B)** Bright-field (top) and epifluorescence (middle and bottom) images of a slice containing the AC injection site for AAV1.GFP.Flex. **(C)** Left: epifluorescence confocal image of a coronal section containing the left AC with Parv tdTomato-positive interneurons. Middle: epifluorescence confocal image of CC-Parv GFP-positive neurons. Right: merged epifluorescence confocal image of Parv tdTomato-positive interneurons and CC-Parv GFP-positive neurons. Layers are indicated on the right.

**Figure 2:**
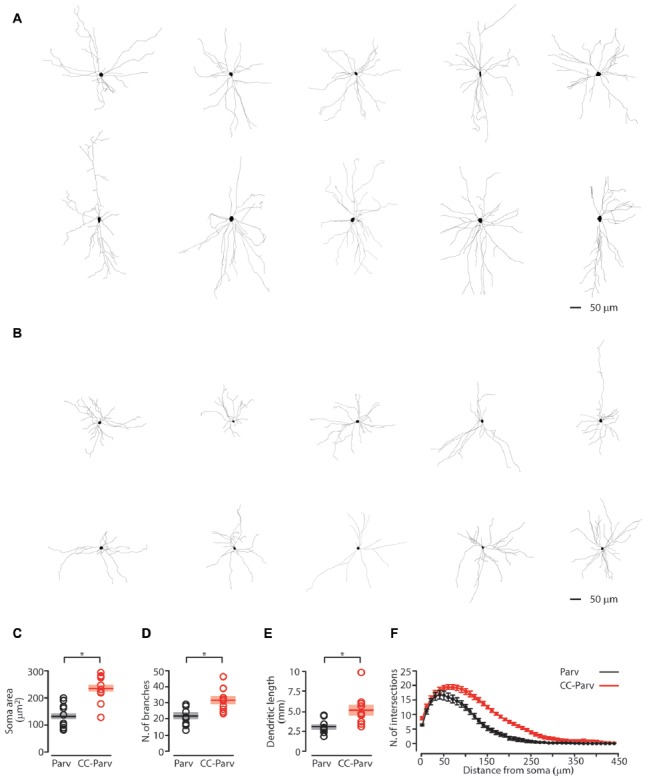
Anatomical characterization of layer 5 Parv and CC-Parv neurons. **(A)** Layer 5 morphological reconstructions of CC-Parv neurons. **(B)** Layer 5 morphological reconstructions of Parv neurons. **(C)** Plot depicting the group average soma area (± S.E.M.) of layer 5 Parv (black) and CC-Parv (red) neurons. Individual circle mark the soma area for each neuron analyzed (layer 5 Parv: black circle, *n* = 18, animals *n* = 10; layer 5 CC-Parv: red circles, *n* = 21, animals *n* = 10). **(D)** Same as in panel **(C)**, for the number of branches. **(E)** Same as in panel **(C)**, for the dendritic length. **(F)** Plot shows the group average Sholl analysis (± S.E.M.) of layer 5 Parv (black) and CC-Parv (red) neurons (layer 5 Parv: black circle, *n* = 18, animals *n* = 10; layer 5 CC-Parv: red circles, *n* = 21, animals *n* = 10); *p < 0.05.

GABAergic projections originating in the cortex and terminating in the contralateral cortex were visualized as previously described (Rock et al., 2017) Briefly, we conditionally expressed GFP in parvalbumin-expressing (Parv) interneurons by injecting AAV1.GFP.Flex into the right AC of Parv-Cre-tdTomato transgenic mice (Figures 1A,B). By using this viral approach, interhemispheric long-range Parv neurons (CC-Parv neurons) were retrogradely labeled with GFP in the contralateral cortex (left AC). GFP was colocalized with Parv/tdTomato-expressing neurons and the somata of CC-Parv neurons were present in all layers of the AC (Figure 1C), allowing identification of the CC-Parv neurons from the rest of the layer 5 parvalbumin-expressing neurons (from this point forward referred to as Parv neurons). Next we tested the hypothesis that layer 5 CC-Parv neurons have different morphological properties compared to the same layer Parv neurons. To this end we analyzed the morphology of biocytin-filled layer 5 CC-Parv (*n* = 10) and Parv (*n* = 10) neurons (Figures 2A,B).

We first quantified the soma area of the layer 5 CC-Parv and Parv neurons. The soma area of layer 5 CC-Parv neurons was significantly larger compared to the layer 5 Parv neurons (layer 5 CC-Parv: 233.9 ± 16.5 μm^2^, *n* = 10; layer 5 Parv: 130.3 ± 14.3 μm^2^, *n* = 10; *p* = 0.0013, rank-sum test; Figure 2C). The diameter of layer 5 CC-Parv neurons was also significantly larger compared to the layer 5 Parv neurons (layer 5 CC-Parv: 17.15 ± 4.3 μm, *n* = 10; layer 5 Parv: 12.71 ± 3.3 μm, *n* = 10; *p* = 1.9 × 10^−4^, *t-test*). Looking at the overall anatomical structure, layer 5 CC-Parv neurons have larger dendritic fields compared to layer 5 Parv neurons. We therefore analyzed the morphological difference between the dendrites of layer 5 CC-Parv and Parv neurons. We measured the number of dendritic branches and found that layer 5 CC-Parv neurons had a larger number of dendritic branches (number of branches layer 5 CC-Parv: 31.4.6 ± 2.2, *n* = 10; number of branches layer 5 Parv: 21.8 ± 1.8, *n* = 10; *p* = 0.006, rank-sum test; Figure 2D). We also found that the total length of dendrites was larger for the layer 5 CC-Parv (dendritic total length layer 5 CC-Parv: 5.186 ± 0.627 mm, *n* = 10; dendritic total length layer 5 Parv: 3.047 ± 0.273 mm, *n* = 10; *p* = 0.002, rank-sum test; Figure 2E). The morphological differences between layer 5 CC-Parv and Parv neurons were confirmed by performing Sholl analysis on individual neurons (Figure 2F).

Having shown that the somatic area and the dendritic morphology of layer 5 CC-Parv and Parv neurons are different, we next decided to compare their functional properties and excitability.

### Electrophysiological Properties of Layer 5 CC-Parv and Parv Neurons in the Mouse AC

**Figure 3:**
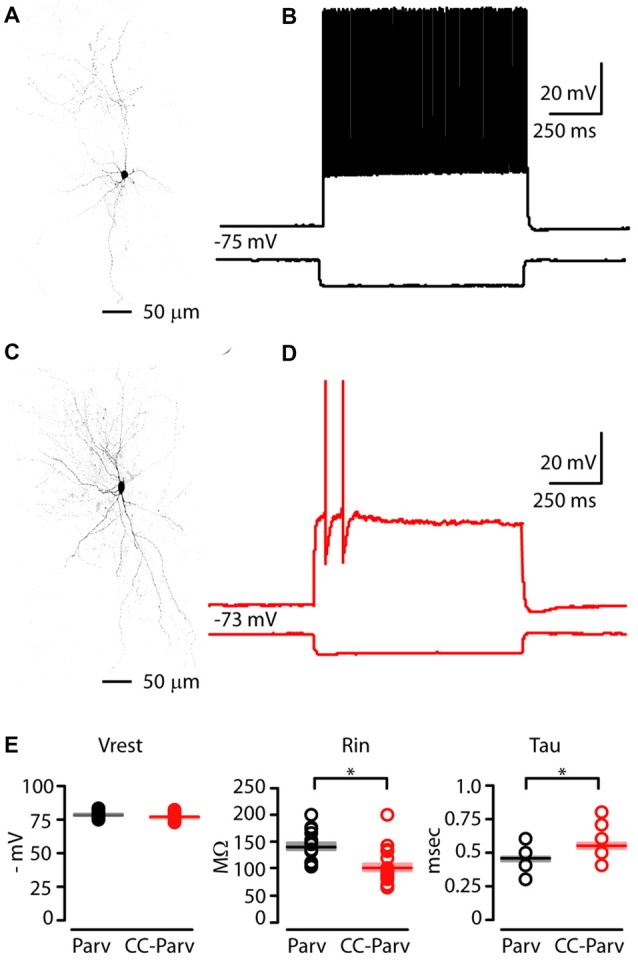
Electrophysiological properties of layer 5 Parv and CC-Parv neurons in the mouse AC. **(A)** High resolution image of a biocytin-labeled layer 5 Parv neuron. **(B)** Response recorded from a layer 5 Parv neuron in the AC during injection of a hyperpolarizing current (1 s −100 pA pulse) and a train of action potentials (APs) recorded during injection of a depolarizing current (1 s 400 pA pulse; black traces). **(C)** High resolution image of a biocytin-labeled layer 5 CC-Parv neuron. **(D)** Same as in panel **(C)**, for the layer 5 CC-Parv neuron (red traces). **(E)** Summary plot of V_rest_: resting membrane potential; R_in_: input resistance; Tau: membrane time constant; recorded from layer 5 Parv (black circles, *n* = 18; animals *n* = 10) and CC-Parv (red circles, *n* = 21; animals *n* = 10) neurons, including group averages (± S.E.M.); *p < 0.05.

In order to identify CC-Parv neurons in the left AC, we used a viral retrograde labeling approach (Figures 1A,B). Thus, viral retrograde labeling allowed us to distinguish and record from visually–identified layer 5 Parv (tdTomato positive) and CC-Parv (GFP/tdTomato positive) neurons in the left AC (Figures 3A,C). Layer 5 Parv neurons exhibited high-frequency firing during prolonged current steps, while layer 5 CC-Parv neurons exhibited low-frequency firing of APs. Figures 3B,D show examples of sub- and suprathreshold responses to current steps recorded from layer 5 Parv and CC-Parv neurons. Additional measurement of the intrinsic electrophysiological properties indicated that layer 5 Parv neurons were resting at the same resting membrane potential (layer 5 Parv: −78 ± 0.6 mV, *n* = 18; layer 5 CC-Parv: −76.5 ± 0.5 mV, *n* = 21; *p* = 0.12, *t*-test), had a higher input resistance (layer 5 Parv: 140.15 ± 6.69 MΩ, *n* = 18; layer 5 CC-Parv: 101.26 ± 6.97 MΩ, *n* = 21; *p* = 3.03 × 10^−5^, *t-test*), and had a faster membrane time constant (layer 5 Parv: 0.45 ± 0.01 ms, *n* = 18; layer 5 CC-Parv: 0.54 ± 0.02 ms, *n* = 21; *p* = 0.0016, *t*-test) compared to layer 5 CC-Parv neurons (Figure 3E).

**Figure 4:**
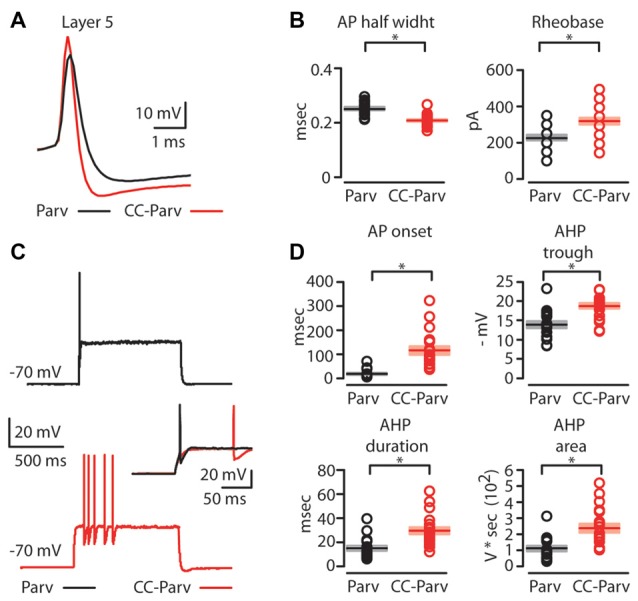
AP properties of layer 5 Parv and CC-Parv neurons.** (A)** Example of an AP recorded from a layer 5 Parv (black trace) and CC-Parv (red trace) neurons. **(B)** Summary plot of AP half-width: AP half-width (left), and Rheobase: the smallest current step evoking an AP (right); recorded from layer 5 Parv (black circles, *n* = 18; animals *n* = 10) and CC-Parv (red circles, *n* = 21; animals *n* = 10) neurons, including group averages (± S.E.M.). **(C)** Representative firing of layer 5 Parv (black trace) and CC-Parv (red trace) neurons in response to near-threshold current step. Inset AP onset latencies recorded in layer 5 Parv (black trace) and CC-Parv (red trace) neurons.** (D)** Top: summary plot of AP onset (left), and afterhyperpolarization (AHP) trough (right); recorded from layer 5 Parv (black circles, *n* = 18; animals *n* = 10) and CC-Parv (red circles, *n* = 21; animals *n* = 10) neurons, including group averages (± S.E.M.). Bottom: summary plot of AHP duration (left), and AHP area (right); recorded from layer 5 Parv (black circles, *n* = 18; animals *n* = 10) and CC-Parv (red circles, *n* = 21; animals *n* = 10) neurons, including group averages (± S.E.M.); *p < 0.05.

Next, we compared the firing properties of layer 5 CC-Parv and Parv neurons near-threshold (Figure 4A). Layer 5 CC-Parv neurons had a shorter AP half-width (layer 5 CC-Parv: 0.21 ± 0.04 ms, *n* = 21; layer 5 Parv: 0.25 ± 0.05 ms, *n* = 18; *p* = 4.6 × 10^−5^, *t*-test), and higher firing threshold (layer 5 CC-Parv: 328.6 ± 20.9 pA, *n* = 21; layer 5 Parv: 230.6 ± 17.2 pA, *n* = 18; *p* = 0.0012, *t*-test), compared to layer 5 Parv neurons (Figure 4B). Figure 4C shows an example of layer 5 Parv (top: black trace) and CC-Parv (bottom: red trace) neurons’ suprathreshold responses to rheobase current steps. The inset shows the first AP delay for Parv (black trace) and CC-Parv neurons (red trace) response to the rheobase current steps (layer 5 CC-Parv: 114.8 ± 17.3 ms, *n* = 21; layer 5 Parv: 17.6 ± 4.1 ms, *n* = 18; *p* = 1.06 × 10^−6^, *rank-sum test*; Figure 4D). The layer 5 CC-Parv neurons also had a larger AHP trough (layer 5 CC-Parv: 18.7 ± 0.7 pA, *n* = 21; layer 5 Parv: 13.8 ± 0.9 pA, *n* = 18; *p* = 5.5 × 10^−6^, *rank-sum test*), duration (layer 5 CC-Parv: 29.4 ± 3 ms, *n* = 21; layer 5 Parv: 14.7 ± 2.1 ms, *n* = 18; *p* = 2.4 × 10^−4^, *rank-sum test*), and area (layer 5 CC-Parv: 235.1 ± 28.6 V * seconds, *n* = 21; layer 5 Parv: 110.2 ± 17.1 V * seconds, *n* = 18; *p* = 2.7 × 10^−4^, *rank-sum test*; Figure 4D).

**Figure 5:**
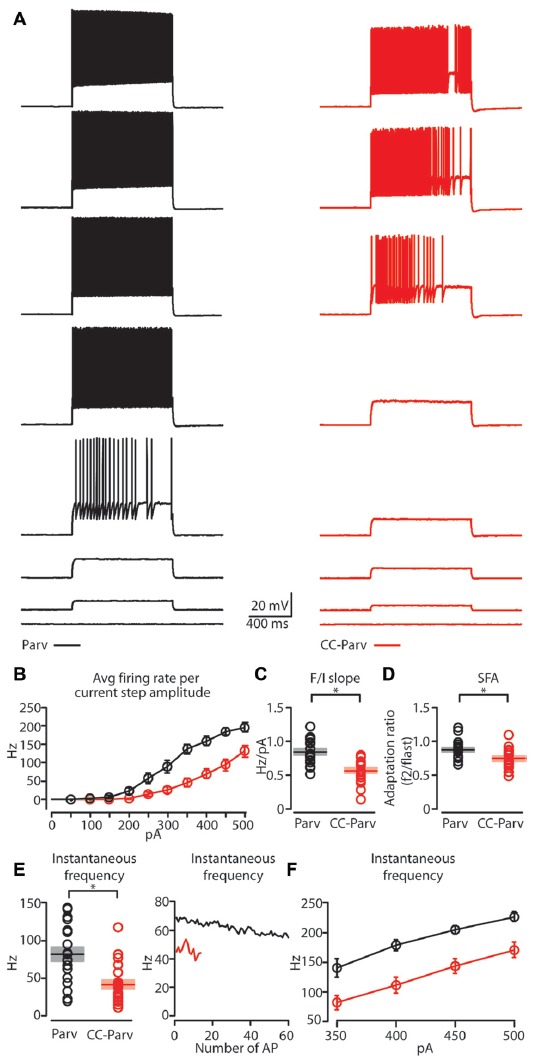
Repetitive firing properties of layer 5 Parv and CC-Parv neurons. **(A)** Representative firing of layer 5 Parv (black traces) and CC-Parv (red traces) neurons in response to increasing depolarizing current (0–350 pA, 50 pA increments). **(B)** Summary plot of *averaging firing rate per current step amplitude* recorded from layer 5 Parv (black circles, *n* = 18; animals *n* = 10) and CC-Parv (red circles, *n* = 21; animals *n* = 10) neurons, including group averages (± S.E.M.).** (C)** Same as in panel **(B)**, for F/I slope.** (D)** Same as in panel **(B)**, for *spike frequency adaptation (SFA; f*_2_/*f*_last_). **(E)** Left: summary plot of instantaneous frequency near-treshold from layer 5 Parv (black circles, *n* = 18; animals *n* = 10) and CC-Parv (red circles, *n* = 21; animals *n* = 10) neurons, including group averages (± S.E.M.). Right: representative instantaneous frequency near-threshold as a function of number of AP from layer 5 Parv (black trace) and CC-Parv (red trace). **(F)** Summary plot of instantaneous firing frequency in response to increasing depolarizing current (350–500 pA, 50 pA increments) for layer 5 Parv (black circles, *n* = 18; animals *n* = 10) and CC-Parv (red circles, *n* = 21; animals *n* = 10) neurons, including group averages (± S.E.M.); *p < 0.05.

Next, we compared the repetitive firing properties of layer 5 Parv (Figure 5A, black traces) and CC-Parv neurons (Figure 5A, red traces). The repetitive firing patterns of layer 5 CC-Parv and Parv neurons differed in their response to depolarizing currents. Layer 5 CC-Parv neurons average firing rate per current step amplitude, and F/I slope (layer 5 CC-Parv: 0.55 ± 0.04 Hz/pA, *n* = 21; layer 5 Parv: 0.84 ± 0.04 Hz/pA, *n* = 18; *p* = 2.2 × 10^−5^, *t*-test) was lower than layer 5 Parv neurons, while layer 5 CC-Parv spike frequency adaptation (SFA; layer 5 CC-Parv: 0.73 ± 0.03, *n* = 21; layer 5 Parv: 0.86 ± 0.03, *n* = 18; *p* = 0.01, *t-test*) was higher than layer 5 Parv neurons (Figures 5B–D).

The initial instantaneous frequency of layer 5 CC-Parv neurons was lower both at current steps near-threshold (layer 5 CC-Parv: 42.8 ± 6.1 Hz, *n* = 21; layer 5 Parv: 81.8 ± 9.4 Hz, *n* = 18; *p* = 0.002, *rank-sum test*), and in response to higher depolarizing currents (layer 5 CC-Parv, I-step 350–500 pA: 81.5 ± 12.2 Hz, 110.1 ± 13.4 Hz, 143.1 ± 12.1 Hz, 170.7 ± 13.3 Hz* n* = 21; layer 5 Parv, I-step 350–500 pA: 140.1 ± 15.5 Hz, 178.8 ± 9.3 Hz, 204.4 ± 7.6 Hz, 226.3 ± 7.3 Hz, *n* = 18; *p* = 0.006, *p* = 5.3 × 10^−4^, *p* = 4.4 × 10^−4^, *p* = 0.002, *t*-test; Figures 5E,F). Accordingly, two populations of layer 5 parvalbumin-expressing neurons of AC can be differentiated on the basis of distinct intrinsic electrophysiological properties.

### Do K_v_ Channels Dampen the Excitability of Layer 5 CC-Parv Neurons?

The finding that layer 5 CC-Parv neurons are characterized by a strong AHP and delayed firing suggested the possibility that the slow inactivation of K^+^ current can be responsible for controlling the near-threshold excitability of CC-Parv neurons (Figure 4). The kinetic and voltage-dependent properties of Kv1 K^+^ channels are heterogeneous (Rudy et al., 2009); those known to be activated near-threshold potentials are characterized by subunits of the Kv1 sub-family that display differences in inactivation as a function of the precise subunit composition (Coetzee et al., 1999). For this reason, we considered Kv1 channels to be strong candidates to explain the mechanism in question.

**Figure 6:**
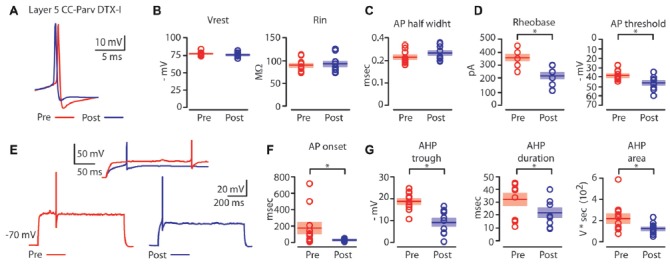
Kv1 channels affects AP properties of layer 5 CC-Parv neurons.** (A)** Example of AP recorded from a layer 5 CC-Parv neurons before (red trace) and after (blue trace) bath application if DTX-I.** (B)** Summary plot of V_rest_ (left): resting membrane potential; R_in_ (right): input resistance; recorded from layer 5 CC-Parv neurons before (red circles, *n* = 10; animals *n* = 5) and after (blue circles, *n* = 10; animals *n* = 5) bath application if DTX-I, including group averages (± S.E.M.). **(C)** Same as in panel **(B)**, for AP half width.** (D)** Summary plot of Rheobase (left), and AP threshold (right); recorded from layer 5 CC-Parv neurons pre- (black circles, *n* = 10; animals *n* = 5) and post- (blue circles, *n* = 10; animals *n* = 5) DTX-I, including group averages (± S.E.M.). **(E)** Representative firing in response to near-threshold current step of layer 5 CC-Parv neuron before (red trace) and after (blue trace) bath application of DTX-I. Inset AP onset latencies recorded in layer 5 CC-Parv neuron pre- (red trace) and post- (blue trace) DTX-I bath application.** (F)** Summary plot of AP onset recorded from layer 5 CC-Parv neurons pre- (red circles, *n* = 10; animals *n* = 5) and post- (blue circles, *n* = 10; animals *n* = 5) DTX-I, including group averages (± S.E.M.). **(G)** Summary plot of AHP trough (left); AHP duration (middle); and AHP area (right) recorded from layer 5 CC-Parv neurons before (red circles, *n* = 10; animals *n* = 5) and after (blue circles, *n* = 10; animals *n* = 5) bath application of DTX-I, including group averages (± S.E.M.); *p < 0.05.

To test this hypothesis, we recorded APs in whole-cell mode from viral retrogradely labeled CC-Parv neurons in the absence and presence of the Kv1-specific peptide toxin dendrotoxin-I (DTX-I) K^+^ channel blockers (Figure 6). DTX-I is a specific antagonist of channels containing Kv1.1, −1.2, and/or 1.6 subunit. These sub-units are the most expressed Kv1 channels of the neocortex (Castle et al., 1994; Coetzee et al., 1999).

We compared the near-threshold AP properties of layer 5 CC-Parv neurons before (Figure 6A, red trace) and after (Figure 6A, blue trace) bath application of DTX-I (50–100 nM). DTX-I had no effect on CC-Parv resting membrane potential (layer 5 CC-Parv pre-DTX-I: −77.1 ± 0.9 mV; layer 5 CC-Parv post-DTX-I: −75.9 ± 1.1 mV;* n* = 11), and input resistance (layer 5 CC-Parv pre-DTX-I: 89.5 ± 4.1 MΩ; layer 5 CC-Parv post-DTX-I: 92.7 ± 5.3 MΩ;* n* = 11; Figure 6B). These data suggest that at rest there is a little or no DTX-I sensitive active current. Similarly, bath application of DTX-I also had no effect on AP half-width (layer 5 CC-Parv pre-DTX-I: 0.21 ± 0.08 ms, *n* = 10; layer 5 Parv post-DTX-I: 0.23 ± 0.09 ms, *n* = 10; Figure 6C). However, DTX-I produced a decrease in the rheobase (layer 5 CC-Parv pre-DTX-I: 359.1 ± 22.1 pA, *n* = 10; layer 5 Parv post-DTX-I: 218.2 ± 21.6 pA, *n* = 10;* p* = 1.9 × 10^−4^, *t*-test), and AP threshold (layer 5 CC-Parv pre-DTX-I: −38.5 ± 1.6 mV, *n* = 10; layer 5 Parv post-DTX-I: −46.2 ± 2.1 mV, *n* = 10;* p* = 0.009, *t*-test; Figure 6D). In addition, DTX-I abolished the delayed firing of layer 5 CC-Parv neurons (layer 5 CC-Parv pre-DTX-I: 180.2 ± 68.3 ms, *n* = 10; layer 5 Parv post-DTX-I: 29.9 ± 4.0 ms, *n* = 10;* p* = 0.03, *t*-test; Figures 6E,F), and decreased the AHP trough (layer 5 CC-Parv pre-DTX-I: 18.8 ± 1.2 mV, *n* = 10; layer 5 CC-Parv post-DTX-I: 9.3 ± 1.6 mV, *n* = 10; *p* = 6.4 × 10^−4^, *rank-sum test*), duration (layer 5 CC-Parv pre-DTX-I: 32.6 ± 4.7 ms, *n* = 10; layer 5 CC-Parv post-DTX-I: 22.2 ± 3.5 ms, *n* = 10; *p* = 0.03 *t*-test), and area (layer 5 CC-Parv pre-DTX-I: 223.9 ± 42.9 V * seconds, *n* = 10; layer 5 CC-Parv post-DTX-I: 126.2 ± 15.1 V * seconds, *n* = 10; *p* = 0.04, *t-test*; Figure 6G).

**Figure 7:**
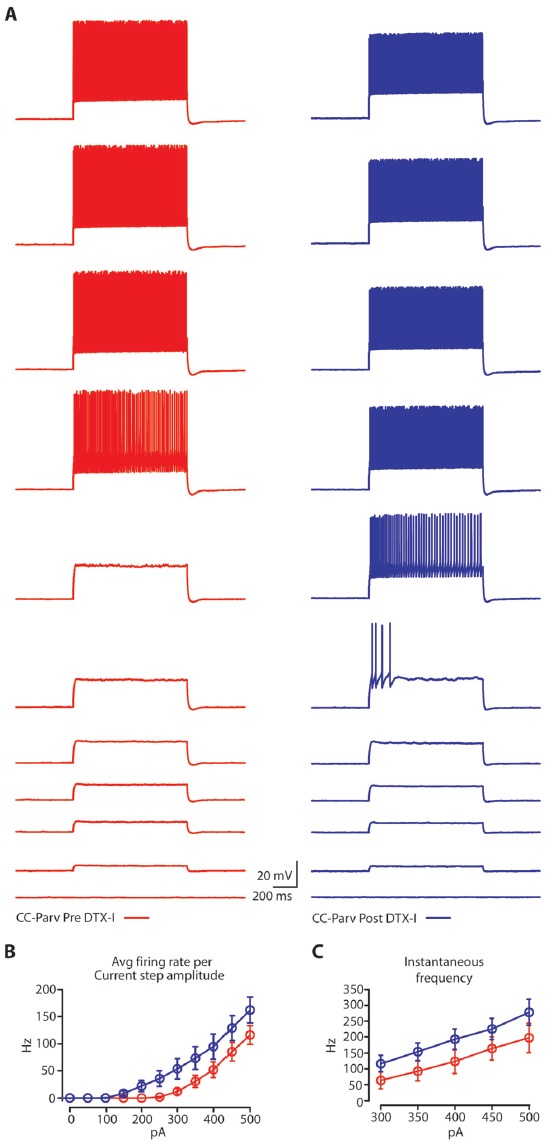
Regulation of layer 5 CC-Parv neurons repetitive firing properties by bath application of DTX-I.** (A)** Representative firing of layer neurons in response to increasing depolarizing current (0–500 pA, 50 pA increments), before (red trace) and after (blue trace) bath application if DTX-I. **(B)** Summary plot of *averaging firing rate per current step amplitude* in response to increasing depolarizing current (0–500 pA, 50 pA increments) recorded from layer 5 CC-Parv before (red circles, *n* = 10; animals *n* = 5) and after (blue circles, *n* = 10; animals *n* = 5) bath application if DTX-I, including group averages (± S.E.M.). **(C)** Summary plot of instantaneous firing frequency in response to increasing depolarizing current (300–500 pA, 50 pA increments) for layer 5 CC-Parv before (red circles, *n* = 10; animals *n* = 5) and after (blue circles, *n* = 10; animals *n* = 5) bath application if DTX-I, including group averages (± S.E.M.).

Next, we tested the effect of DTX-I on the repetitive firing properties of layer 5 CC-Parv (Figure 7A left, red traces pre-DTX-I; Figure 7B right, blue traces post-DTX-I). Both the average firing rate per current step amplitude and the instantaneous frequency were higher after blockade of Kv1 channels by bath application of DTX-I (Figures 7B,C).

These data reveal that Kv1 channels are the main sources of the repolarization of the layer 5 CC-Parv AP and have a major role in determining both the spike delayed response, frequency and instantaneous frequency of these neurons.

### Are Kv1.1 Containing K^+^ Channels Responsible for Regulating the Excitability of CC-Parv Neurons?

**Figure 8:**
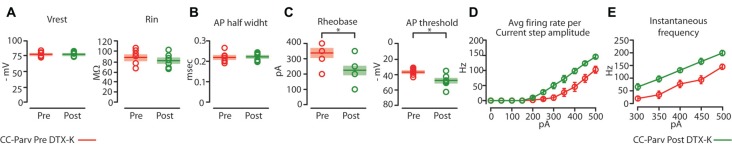
Kv1.1 containing K^+^ channels regulate the excitability of CC-Parv neurons. **(A)** Summary plot of V_rest_ (left): resting membrane potential; R_in_ (right): input resistance; recorded from layer 5 CC-Parv neurons before (red circles, *n* = 7; animals *n* = 5) and after (green circles, *n* = 7; animals *n* = 5) bath application if DTX-KI, including group averages (± S.E.M.). **(B)** Same as in panel **(A)**, for AP half width. **(C)** Summary plot of Rheobase (left), and AP threshold (right); recorded from layer 5 CC-Parv neurons pre- (red circles, *n* = 7; animals *n* = 5) and post- (green circles, *n* = 7; animals *n* = 5) DTX-K, including group averages (± S.E.M.). **(D)** Summary plot of *averaging firing rate per current step amplitude* in response to increasing depolarizing current (0–500 pA, 50 pA increments) recorded from layer 5 CC-Parv before (red circles, *n* = 7; animals *n* = 5) and after (green circles, *n* = 7; animals *n* = 5) bath application if DTX-K, including group averages (± S.E.M.). **(E)** Summary plot of instantaneous firing frequency in response to increasing depolarizing current (300–500 pA, 50 pA increments) for layer 5 CC-Parv before (red circles, *n* = 10; animals *n* = 5) and after (Green circles, *n* = 7; animals *n* = 5) bath application if DTX-K, including group averages (± S.E.M.); *p < 0.05.

Next, we recorded APs in whole-cell mode, from viral retrogradely labeled CC-Parv neuron in the absence and presence of the Kv1-specific peptide toxin dendrotoxin-K (DTX-K) K^+^ channel blockers (Figure 8). As DTX-K is a Kv1.1-specific blocker, pharmacological blockade allowed us to assess the contribution of this Kv1-specific subunit to the excitability of CC-Parv neurons (Robertson et al., 1996; Goldberg et al., 2005, 2008; Kole et al., 2007).

**Figure 9:**
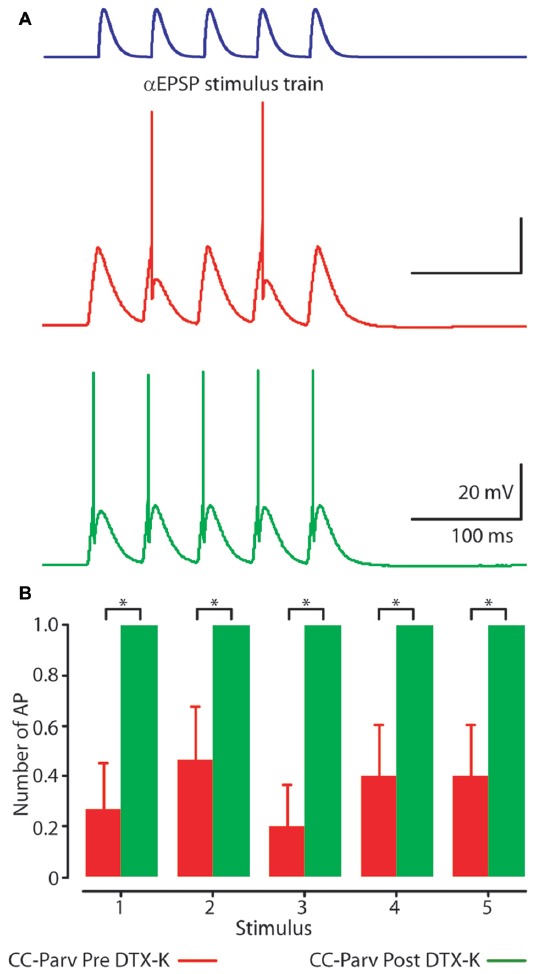
Kv1.1 containing K^+^ channels shape layer 5 CC-Parv neurons firing.** (A)** Example responses to a 20 Hz train of 5 EPSC-like waveforms (top) recorded from layer 5 CC-Parv neuron before (red trace), and after (green trace) bath application of DTX-K. Layer 5 CC-Parv neurons show increased firing after bath application of DTX-K (green trace). **(B)** Average plot of AP responses to a train of EPSC-like waveforms before (red bars), and after (green bars) bath application of DTX-K, including group averages (± S.E.M.); *p < 0.05.

Perfusion of slices with 50–100 nM DTX-K had no effect on CC-Parv resting membrane potential (layer 5 CC-Parv pre-DTX-K: −77.4 ± 1.6 mV, *n* = 7; layer 5 CC-Parv post-DTX-K: −77.5 ± 1.6 mV;* n* = 7), and input resistance (layer 5 CC-Parv pre-DTX-K: 86.7 ± 5.5 MΩ, *n* = 7; layer 5 CC-Parv post-DTX-K: 81.2 ± 5.1 MΩ;* n* = 7; Figure 8A). In line with the nonspecific DTX-I blockade, the use of the DTX-K antagonist indicates that little to no Kv1.1 current is active at rest. Similarly, bath application of DTX-K had no effect on AP half-width (layer 5 CC-Parv pre-DTX-K: 0.21 ± 0.09 ms, *n* = 7; layer 5 Parv post-DTX-K: 0.22 ± 0.07 ms, *n* = 7; Figure 8B). However, DTX-K produced a decrease in the rheobase (layer 5 CC-Parv pre-DTX-KI: 335.7 ± 28.3 pA, *n* = 7; layer 5 Parv post-DTX-KI: 221.4 ± 28.5 pA, *n* = 7;* p* = 0.014, *t*-test), and AP threshold (layer 5 CC-Parv pre-DTX-K: −36.8 ± 1.6 mV, *n* = 7; layer 5 Parv post-DTX-KI: −48.1 ± 3.1 mV, *n* = 7;* p* = 0.007, *t*-test; Figure 8C). In addition, DTX-K abolished the delayed firing of layer 5 CC-Parv neurons, and decreased the AHP trough duration and area (data not shown). Next, we tested the effect of DTX-K in determining the repetitive firing properties of layer 5 CC-Parv. The average firing rate per current step amplitude and instantaneous frequency of layer 5 CC-Parv neurons were higher after the bath application of DTX-K (Figures 8D,E). These results indicate that differences in levels of Kv1.1 containing K^+^ channels current should dynamically regulate the excitability of layer 5 CC-Parv neurons. To further test this, we injected a train of 5 EPSP-like waveforms at 20 Hz (αEPSPs) into the soma via the patch pipette (Figure 9A). We found that after a bath application of DTX-K, layer 5 CC-Parv neurons showed a marked increase firing responses to the 5 EPSP-like waveforms (Figures 9A,B).

Overall, these data show that Kv1.1 containing K^+^ channels are responsible for regulating the excitability of layer 5 CC-Parv neurons.

## Discussion

In this study we demonstrate that the layer 5 parvalbumin-expressing neurons with inhibitory callosal projection present distinct anatomical and excitability properties in the mouse AC. Additionally, our results conclude that the Kv1.1 containing K^+^ channels regulate the excitability of layer 5 CC-Parv neurons. As we described previously (Rock et al., 2017), the employed approach (limited injection volume and variability in transfection leading to partial coverage of the cortical area) precludes us from determining the absolute number of anatomical and electrophysiological differences of layer 5 long-range CC-Parv vs. short-range Parv neurons. However, our viral retrograde labeling approach allowed us to routinely determine the dendritic morphology and electrophysiological properties of layer 5 long-range parvalbumin-expressing neurons and establish the presence of two distinct subclasses of GABAergic neurons.

### Layer 5 CC-Parv and Parv Neurons: From Dendritic Field to Response Selectivity

In rodent brains GABAergic neurons represent only 10%–15% of the entire cortical neuronal population and are the primary source of inhibition (Meyer et al., 2011). This class of neurons is composed of molecularly and morphologically heterogeneous subgroups (for review see, Blatow et al., 2005; Ascoli et al., 2008; Xu et al., 2010; Rudy et al., 2011; Caputi et al., 2013; DeFelipe et al., 2013; Tremblay et al., 2016). The seminal studies by Ramon y Cajal established the presence of a large number of cells with “short-axons” in the brain (Ramon Y Cajal et al., 1988). These GABAergic neurons have been considered to project locally in the cortex, and for this reason were described with the word “interneurons”. However, recent studies (Melzer et al., 2012, 2017; Lee et al., 2014; Tomioka et al., 2015; Basu et al., 2016; Rock et al., 2016, 2017) have demonstrated that long-range GABAergic projections may be more prevalent than previously assumed. One of the main findings of the present study is that layer 5 CC-Parv neurons had larger dendritic fields characterized by longer dendrites that branched farther from the soma, whereas layer 5 Parv neurons had smaller dendritic fields characterized by shorter dendrites that branched nearer to the soma.

Parvalbumin-expressing neurons have been shown to receive input from different pathways (Helmstaedter et al., 2009; Xu and Callaway, 2009; Bagnall et al., 2011; Kubota et al., 2011; Tukker et al., 2013). In addition, several studies have examined the membrane potential dynamics and receptive field of GABAergic interneurons (Niell and Stryker, 2008; Liu et al., 2009, 2010; Gentet et al., 2010; Kerlin et al., 2010; Runyan et al., 2010; Hamilton et al., 2013; Li et al., 2015; Resnik and Polley, 2017) and have described the existence of both broadly and highly tuned neurons. Our findings, together with a previous study from Runyan and Sur (2013), in which the response selectivity of layer 2/3 parvalbumin-expressing neurons were found to correlate with dendritic morphology, invite us to speculate that layer 5 CC-Parv and Parv neurons have different receptive fields in response to sound stimuli.

Based on our morphological data, layer 5 CC-Parv-neurons may be broadly tuned due to a larger dendritic field, while layer 5 Parv neurons would be highly tuned due to a smaller dendritic field, suggesting that these two specific subtypes of layer 5 parvalbumin-expressing neurons likely perform specific cortical functions (Markram et al., 2004; Somogyi and Klausberger, 2005; Isaacson and Scanziani, 2011; Tremblay et al., 2016).

### Excitability Properties of Layer 5 CC-Parv and Parv Neurons

In this study, we report not only anatomical differences between layer 5 CC-Parv and Parv neurons, but also distinct electrophysiological properties of these two parvalbumin-expressing neurons that are likely to contribute to the function of these neurons with the cortical circuits.

We found that layer 5 CC-Parv neurons are characterized by a delayed AP response near-threshold, as well as a lower firing rate and instantaneous frequency compared to the layer 5 Parv neurons. Bath application of either of the Kv1-specific blocker DTX-I or the Kv1.1 (for review see, Pongs, 2007; Rudy et al., 2009; Jan and Jan, 2012) subunit-specific blocker DTX-K abolished the characteristic delayed-response of the first AP observed near threshold current injections, converting the firing pattern to a more continuous discharge pattern as was observed for the layer 5 Parv neurons. By blocking this current, a large reduction in both the threshold current injection and the voltage threshold for the generation of the AP was observed that ultimately increased the instantaneous frequency in the layer 5 CC-Parv neurons. Overall, our study suggests that Kv1.1 containing K^+^ channels are the main sources of the AP repolarization of the layer 5 CC-Parv and have a major role in determining both the spike delayed response, firing rate and instantaneous frequency of these neurons. However, our study does not exclude the possibility that Kv1.1 containing K^+^ channels is expressed and contributes to the electrophysiological properties of layer 5 Parv neurons.

We conclude that the Kv1 conductance allows the layer 5 CC-Parv neurons to filter out weak and slowly rising inputs in order to allow these neurons to respond to large and synchronous inputs. Particularly, the Kv1.1 containing K^+^ channels dampen the excitability of the layer 5 CC-Parv neurons, serving to gate long-range interhemispheric inhibition. Since the most expressed Kv1 channels of the neocortex (Castle et al., 1994; Coetzee et al., 1999) are composed of Kv1.1, −1.2, and/or 1.6 subunit, future experiments will provide insight about the specificity of the Kv1 subunit composition and localization between the layer 5 CC-Parv and Parv neurons (Kole et al., 2007; Goldberg et al., 2008).

### Layer 5 CC-Parv and Parv Neurons: From Cell-Type to Cortical Dynamic

The physiology underlying different cell-types of GABAergic neurons, as well as their connectivity within the cortex is virtually unknown, and numerous questions about the organization of long-range cortical circuits need to be addressed. An imperative goal is to determine the differences between CC-Parv neurons and short-axon Parv interneurons in generating cortical oscillations locally and across cortical areas. It is well established that callosal projections release the excitatory neurotransmitter glutamate, and serve to merge information between the two hemispheres via excitatory signals. However, more recent findings that the hemispheres can inhibit each other via callosal projections (for review, see Bloom and Hynd, 2005) suggests that callosal function may be more complex. An important unsolved question is aimed at understanding how callosal projections influence the transfer of information, such as global synchronization, between the two hemispheres: what is the contribution of excitation vs. inhibition? Data obtained from our lab indicates that layer 5 CC-Parv and Parv neurons, while expressing the same molecular marker as the corresponding GABAergic neurons with local axonal arborization, have different anatomical and excitability properties when compared (Rock et al., 2016, 2017). Cortical rhythmic activity is a key feature of neuronal activity that has been observed from insects and primates across a wide range of cortical regions (Buzsáki and Draguhn, 2004). In the cortex, synchronization of neuronal activity, such as gamma (30–80 Hz) oscillations has been thought to facilitate communication between pyramidal neurons across brain areas. Particularly, the circuits underlying gamma oscillations are thought to depend entirely on Parv neurons with local axonal arborization (Buzsáki and Wang, 2012). As pointed out by Buzsáki et al. (2004) functional diversity of GABAergic neurons is ideal for enhancing the computational power of cortical circuits at a low wiring cost (GABAergic neurons with local axonal arborization).

However, there is a trade-off between cortical synchronization and wiring economy. Particularly, Buzsáki et al. (2004) demonstrated that a network of GABAergic neurons made of parvalbumin-expressing neurons with local axonal arborization mainly generate a form of transient local synchronization without global synchronization. The addition of GABAergic neurons with long-range axonal arborization significantly increased global synchronization as was evidenced by the emergence of a clear oscillation. Both the passive and active membrane properties of the neurons can determine their role in a circuit; our data indicate that GABAergic neurons with long-range axonal arborization can behave differentially in the context of cortical circuits. For example, CC-Parv would not respond to a weak stimulus, but would participate in the circuit in response to a strong stimulus by inhibiting its postsynaptic targets. Together with other CC-Parv neurons, CC-Parv would impose a rhythmic oscillation that is dependent and scales up with stimulus intensity. In contrast, Parv neurons would participate in feedforward and feedback inhibition in response to weak stimuli. Despite theoretical and computer modeling studies showing that GABAergic neurons with long-range axonal arborization are crucial for cortical processing such as synchronization, the importance of each GABAergic cell-type for distinct roles in cortical processing has yet to be determined. Future experiments will provide further insight on the complexity of the anatomical and molecular composition of the long-range GABAergic projections cell-types and their role in cortical processing.

## Author Contributions

AJA: general development of the project, financial support, experimental design, electrophysiology recording and data analysis and writing the manuscript. HZ: data analysis and drafting the manuscript. PLCF: writing the manuscript and analysis of the data.

## Conflict of Interest Statement

The authors declare that the research was conducted in the absence of any commercial or financial relationships that could be construed as a potential conflict of interest. The reviewer GM and handling Editor declared their shared affiliation.

## Footnotes

^1^www.ephus.org
